# Eigenmodes of Latent-Symmetric Quantum Photonic Networks

**DOI:** 10.1021/acsphotonics.5c01082

**Published:** 2025-11-25

**Authors:** Jonas Himmel, Max Ehrhardt, Matthias Heinrich, Malte Röntgen, Alexander Szameit, Tom A. W. Wolterink

**Affiliations:** † Institute of Physics, 9187 University of Rostock, 18059 Rostock, Germany; ‡ Eastern Institute for Advanced Study, Eastern Institute of Technology, Ningbo, Zhejiang 315200, People’s Republic of China

**Keywords:** latent symmetry, integrated photonics, coupled modes, quantum photonics, eigenmodes

## Abstract

We investigate the impact of latent symmetries on the dynamics of photonic systems and their eigenmodes. Residing solely within the eigenspectral domain, latent symmetries are not visible in real space yet promise intriguing new ways to engineer the functionality of photonic systems. We study the eigenmodes of a 9-site latent-symmetric photonic network and experimentally demonstrate that classical antisymmetric excitations of the latent-symmetric sites are fundamentally precluded from populating so-called singlet sites. Since arbitrary extensions of the system at these sites do not break its latent symmetry, antisymmetric excitations cannot leave the initial system, which can be leveraged, e.g., for the storage of information. We expand this approach to multiparticle excitations and theoretically investigate how two-photon quantum excitations behave in our photonic network. We find that both latent symmetry and the presence of singlet sites are preserved in this realm. Overall, latent symmetries introduce a powerful new set of tools to the design of systems with desired functionality on any nanophotonic platform, paving the way for applications in photonic information processing.

## Graphical abstract

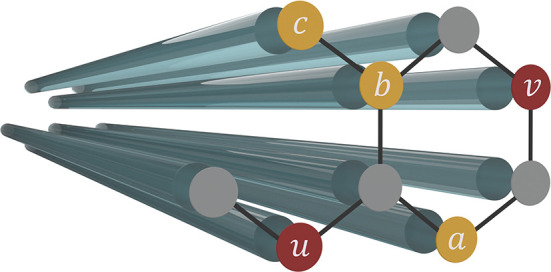

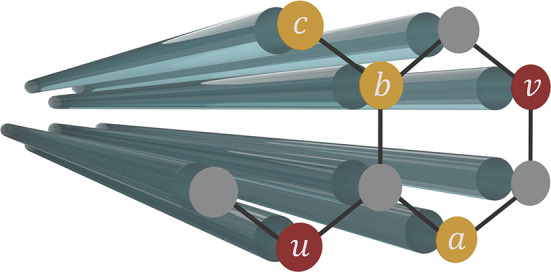

## Introduction

The design and implementation of photonic systems play a key role in photonic information processing, both in the classical and quantum domain. While photonic devices may be realized on a wide variety of technological platforms, ranging from nanophotonic metasurfaces to integrated photonics, most of these structures can be described in terms of coupled modes, which are then tailored to realize the desired functionality. In order to understand and predict the dynamics within such systems, symmetries are of paramount importance. Spatial symmetries, such as permutation symmetry, are well studied in terms of their influence on the system’s dynamics and help to design, e.g., networks for perfect transfer of quantum states.^1–4^ Meanwhile, symmetries in *k*-space serve to establish and classify topological insulators and superconductors,^5^ and *PT*-symmetry can be applied for enhanced sensing^6,7^ or mode-selective laser cavities^8,9^ in non-Hermitian settings. While the presence of such conventional symmetries imposes corresponding symmetry constraints in the spectral domain, spectral symmetries do not necessarily show up in real space yet still influence the behavior of the studied system. This is demonstrated by the concept of cospectrality,^10^ which has been recently introduced in graph theory and gave rise to the concept of so-called latent or “hidden” symmetries.^11,12^ Latent symmetries are not visible in real space and exclusively manifest in the spectrum of the photonic system. They give rise to, e.g., flat bands,^13^ topology,^14^ or non-Hermiticity,^15^ and enable functionalities such as the transfer of states.^16–18^

In this work, we focus on the impact of latent symmetries on the eigenmodes of a photonic system and their effect on the dynamics of single- and two-photon excitations within the system. Starting with single-photon dynamics, we demonstrate experimentally that antisymmetric single-photon excitations of the latent symmetric network sites lead to destructive interference at so-called singlet sites^19^ by implementing an integrated photonic network. Intriguingly, this holds true even when the system is arbitrarily extended to these singlet sites. In other words, an antisymmetric excitation can be “stored” in the initial system without being able to leave it. Expanding this approach to the evolution of distinguishable and indistinguishable two-photon excitations, we show theoretically how the resulting dynamics are influenced by latent symmetry.

## Latent-Symmetric Photonic Systems

To introduce the reader to the exciting field of latent symmetries, let us start by reviewing known results and linking those to the eigenmodes and dynamics of photonic systems. As an example, Figure 1a shows a simple nine-site network. It can be described by a tight-binding Hamiltonian of the form

$$H=\sum_{i}^{N}\beta_{i}|i\rangle \langle i|+\sum_{i,j}^{N}\kappa_{i,j}|i\rangle \langle j|$$

with *N* the number of sites, β_*i*_ the real on-site potential, and κ_*i*,*j*_ the coupling between sites *i* and *j*. In this specific case of Figure 1a, all of the on-site potentials are zero. The coupling between two sites *i* and *j* is either unity when they are connected by a line and zero otherwise. Such tight-binding Hamiltonians can be implemented by photonic systems containing *N*-coupled modes, for example, a network of evanescently coupled waveguides where the time evolution of an initial excitation |ψ­(*t* = 0)⟩ is completely analogous to the propagation of light along the *z*-direction.^20^

**Figure 1:**
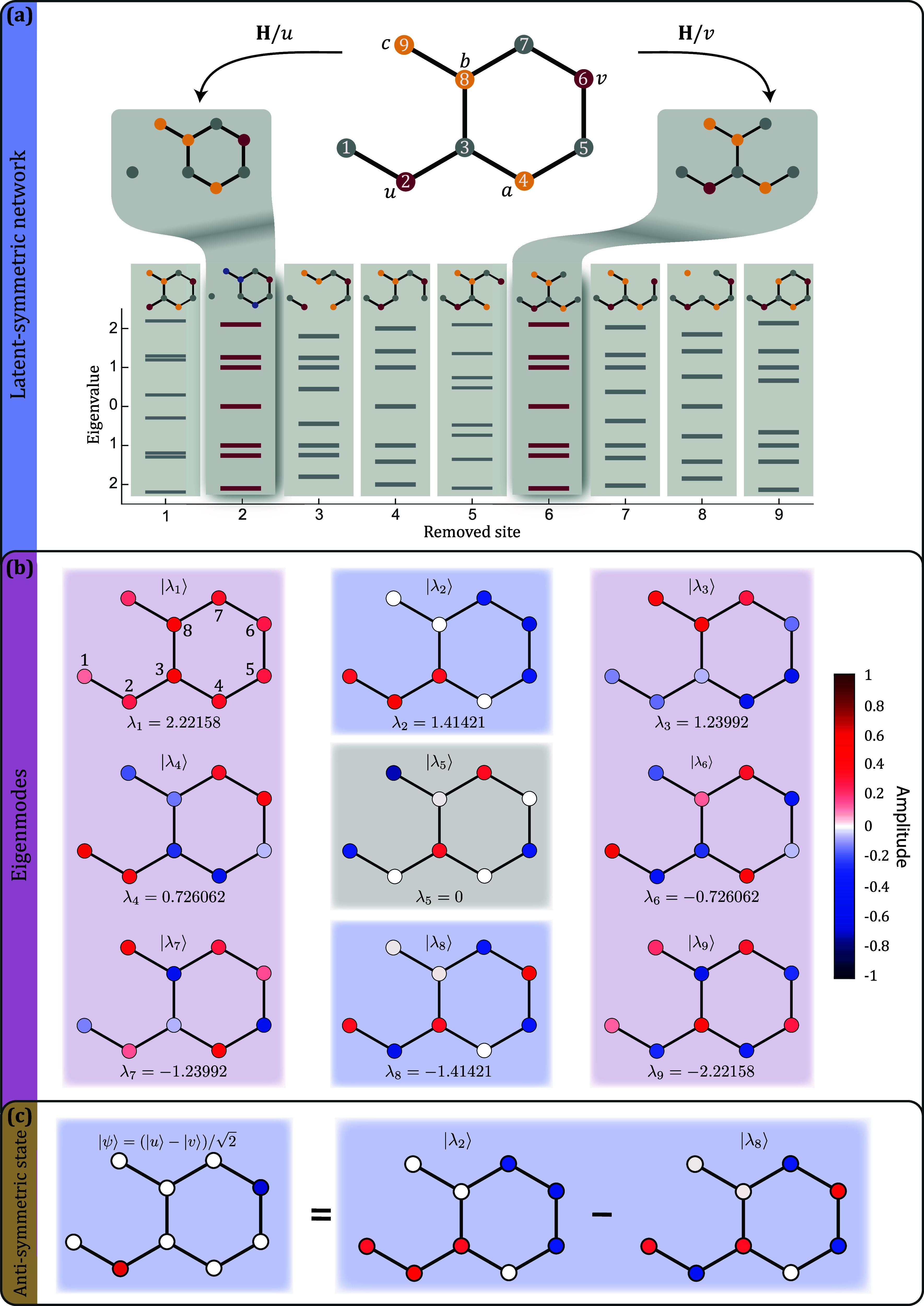
(a) A nine-site system with two latent symmetric sites *u*, *v* and singlet sites *a*, *b*, *c*. While there is no permutation symmetry between *u* and *v*, their respective removal yields two graphs **H**/*u* and **H**/*v* with the same eigenvalue spectrum σ­(**H**/*u*) = (**H**/*v*), and hence the two network sites are latent symmetric. (b) The nine eigenmodes and eigenvalues of the system. The colormap represents the amplitude of the eigenmodes on the respective network site. Sites *u* = 2 and *v* = 6 have definite parity in all modes. Even (odd) parity eigenmodes have red (blue) backgrounds, whereas the eigenmode that vanishes on *u* and *v* has a gray background. (c) An antisymmetric excitation on *u* and *v* only populates the two odd parity eigenmodes, which have vanishing amplitude on the singlet sites *a*, *b*, *c*.

The evolution of an arbitrary initial single-photon excitation |ψ­(*z* = 0)⟩ is then given by

|ψ(z)⟩=U(z)|ψ(z=0)⟩

with the time-evolution operator **U**(*z*) = exp­(−i*z* **H**). A strong link between these dynamics and the systems eigenmodes is found by decomposing the Hamiltonian in terms of its eigenvalues λ_*r*_ and associated eigenmodes |λ_*r*_⟩ to $H=\sum_{r}\lambda_{r}|\lambda_{r}\rangle \langle \lambda_{r}|$ . The time-evolution operator can then be written as

$$U\left(z\right)=\sum_{r}e^{-iz\lambda_{r}}|\lambda_{r}\rangle \langle \lambda_{r}|$$

The dynamics of the system are fully determined by the eigenmodes and eigenvalues and therefore are influenced by symmetries occurring in the spectral regime. One example of such a symmetry is the cospectrality of two network sites. Two sites of a network are considered cospectral if the two networks obtained by removing either one of them share the same set of eigenvalues^10^ (see Figure 1a). While this is obviously the case whenever the two sites are permutation symmetric, this condition can also be fulfilled if they are not. Indeed, two sites can be cospectral without obeying any corresponding symmetry in the spatial domain.^11^ Networks with at least one pair of such sites are termed latent symmetric. In the example shown in Figure 1a, sites *u* = 2 and *v* = 6 exhibit a latent symmetry.

In the absence of degeneracies, all eigenmodes of a latent symmetric network have even or odd parity on the latent symmetric sites *u* and *v*.^10^ Denoting the component of eigenvector |λ_*r*_⟩ on site |*m*⟩ as ⟨λ_*r*_|*m*⟩, this means that ⟨λ_*r*_|*u*⟩ = ±⟨λ_*r*_|*v*⟩ for all eigenvectors. The eigenmodes of the studied network, displayed in Figure 1b, demonstrate this statement. Six of them have even parity on *u* and *v* (red background), two have odd parity (blue background), and one eigenmode has vanishing amplitude on the two sites (gray background). The eigenmodes are ordered by their corresponding eigenvalue with |λ_1_⟩ being the ground mode of the system. Since the system is represented by a bipartite graph, the eigenvalues are symmetric around zero and come in ± pairs,^21^ while the eigenmodes of opposite eigenvalue have same but alternating amplitudes.

## Singlet Sites

Latent symmetric networks may feature one or more so-called singlet sites. A singlet site *w* has the same “distance” to the two cospectral sites *u* and *v* ^19^ and, as a consequence, any odd parity eigenmode can be shown to vanish on *w*. Our system shown in Figure 1a has three singlet sites *a* = 4, *b* = 8, and *c* = 9 (highlighted yellow). As can be seen in Figure 1b, the two odd parity eigenmodes indeed vanish from those sites. We remark that intriguingly, singlet sites allow for arbitrary extension of the network without breaking the latent symmetry between *u* and *v*. Further information on this topic is found in ref 19.

From an experimental perspective, the behavior of antisymmetric single-photon excitations at the two latent-symmetric network sites gives an easily observable signature of latent symmetries. As can be shown, an (anti) symmetric initial excitation in *u* and *v*, $|\psi \rangle =\left(|u\rangle \pm |v\rangle \right)/\sqrt{2}$ , only excites the (odd) even parity eigenmodes of the system.^16^ In Figure 1c, we showcase this statement for an antisymmetric excitation. A particularly intriguing consequence of the above is that an initial antisymmetric excitation in *u* and *v* will vanish on all singlet sites for any propagation distance *z*, because all odd parity eigenmodes have zero amplitude on these sites. This is visualized in Figure 2, where we also visualize the probability as a function of *z* for symmetric excitation. For the antisymmetric excitation, we remark that only two eigenmodes are excited and the time-dependent amplitude at any of the six nonsinglet sites thus follows a cosine behavior.

**Figure 2:**
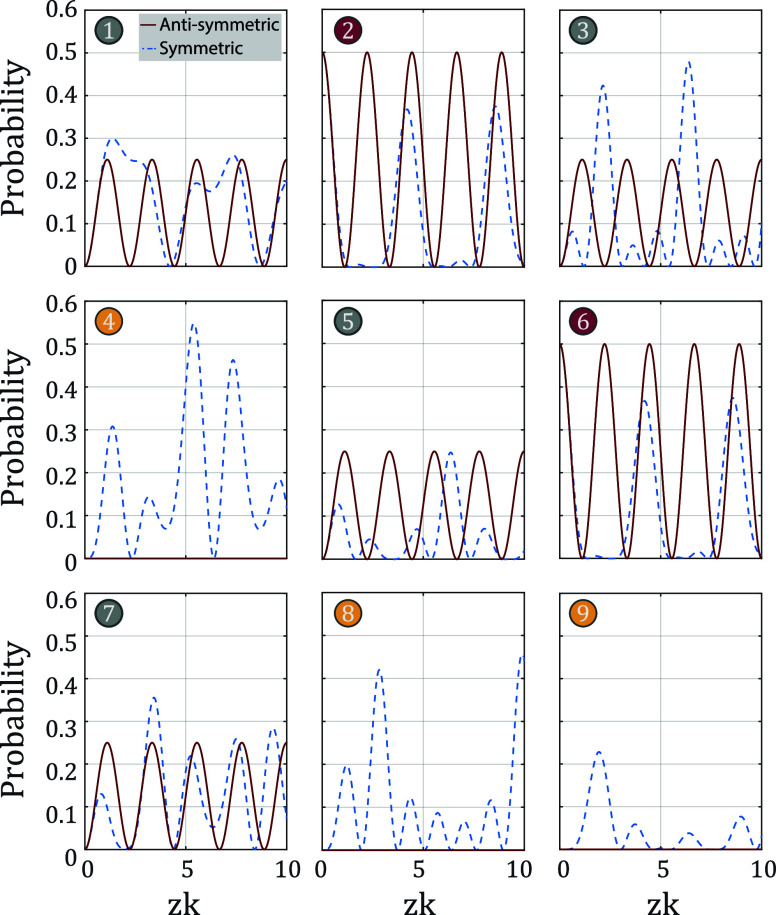
Evolution of a classical symmetric (dashed blue) and antisymmetric (red) initial excitation on the *u* and *v* sites through the latent symmetric photonic network. Shown is the probability as a function of the effective coupling distance defined as the product of propagation distance *z* and coupling constant *k*.

## Results and Discussion

### Experimental Validation of Single-Photon Dynamics

For an experimental validation, we implemented the latent-symmetric network using femtosecond laser-written waveguides.^20^ For the excitation, a fiber coupled to an 814 nm laser diode was used. Note that in our photonic system, the evolution of classical coherent states is mathematically equivalent to the evolution of single photons. A balanced beam splitter and a phase shift of π for one of the two inputs enabled the anti-symmetry of the initial excitation. Switching the input port of the beam splitter leads to symmetric excitation. The end facet of the chip was imaged, using an objective, onto a CMOS camera (Basler ace). The system was written for eight distinct propagation distances ranging from *z* = 82 mm to *z* = 89 mm. The camera image of the end facet is shown in Figure 3a,b for the antisymmetric and symmetric excitation for a propagation distance of *z* = 86 mm. The intensity in all waveguides for all eight propagation distances and both excitations is shown in Figure 3c. The red and dashed blue curves represent the simulated behavior of the ideal network (cf. Figure 2), zoomed into the measured regime of *zk* = 4.4 to 4.8. Figure 3a,b clearly shows that the intensity in singlet sites 4, 8, and 9 remains very close to zero for the antisymmetric excitation, demonstrating the latent symmetry. For the symmetric excitation, the singlet sites do not stay dark.

**Figure 3:**
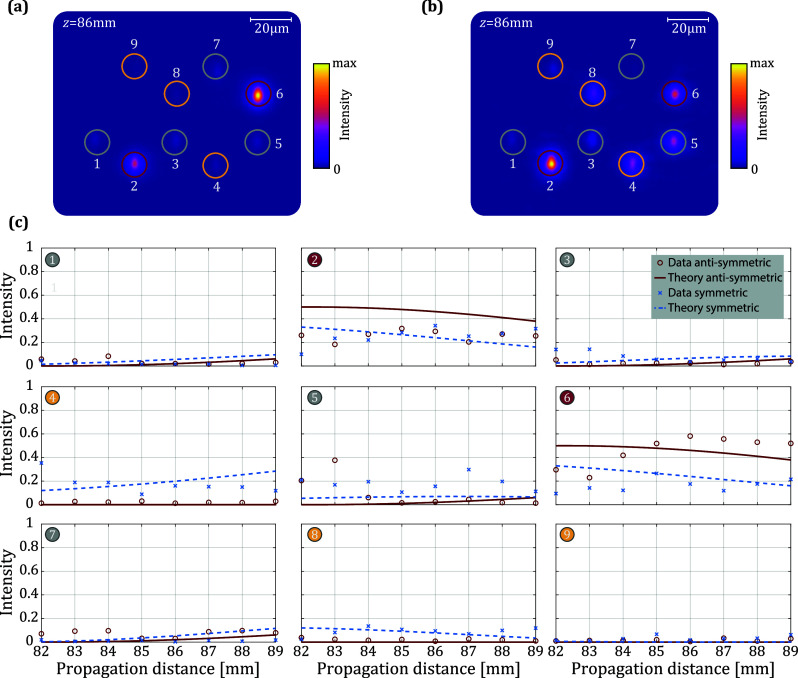
Intensity distribution of the output of the latent-symmetric network for an antisymmetric (a) and symmetric (b) excitation of waveguides 2 and 6 for a propagation distance of *z* = 86 mm. (c) Intensity in the nine waveguides as a function of propagation distances. Measured data for antisymmetric excitation is depicted by red circles, for symmetric excitation by blue crosses. The simulated behavior for the ideal network is depicted by the red and blue line for antisymmetric and symmetric excitation, respectively.

### Distinguishable Two-Photon Excitation

To expand the previous approach and explore the dynamics of a multiparticle excitation, we apply our findings to the evolution of a distinguishable two-photon excitation of the studied system. The dynamics of such an excitation can be described by the two-photon Hamiltonian **H**_Dist_ = **H** ⊕ **H**, where ⊕ denotes the Kronecker sum. **H**_Dist_ corresponds to a network with *N* ^2^ = 81 sites, as depicted in Figure 3a. Each site now represents a two-photon correlation event. Site 32, for example, corresponds to photon one residing at site 5, while photon two is located at site 4. The evolution of a two-photon excitation is then given by the unitary operator $U_{Dist}=e^{-i{zH}_{Dist}}$ . Again, it is determined by the interference of its eigenmodes and thus their symmetries. If Λ is the diagonal matrix containing the single-photon eigenvalues λ_*r*_, then the two-photon eigenvalues are obtained via the diagonal entries of Λ_Dist_ = Λ ⊕ Λ. Similarly, one obtains the eigenmodes of the two-photon Hamiltonian via the matrix *V*_Dist_ = *V* ⊗ *V*, if *V* is the matrix with the single-photon eigenmodes |λ_*r*_⟩ as columns, with ⊗ the Kronecker product.

The resulting Hamiltonian **H**_Dist_ (Figure 4a) features two latent-symmetric pairs and nine singlet sites. The first latent symmetric pair (red) refers to the case of both photons residing at site 2 or both at site 6 of single-photon graph **H**. The second pair (green) refers to one photon being at site 2, the other one at site 6 of **H** and vice versa. The singlet sites of **H**_Dist_ refer to the cases of both photons being at (possibly different) singlet sites of **H**, so to the correlation of both photons being at one of the singlet sites *a*, *b*, or *c*. The sites corresponding to one photon being at a singlet site and one photon at another site of **H** do not become singlet sites of **H**_Dist_.

**Figure 4:**
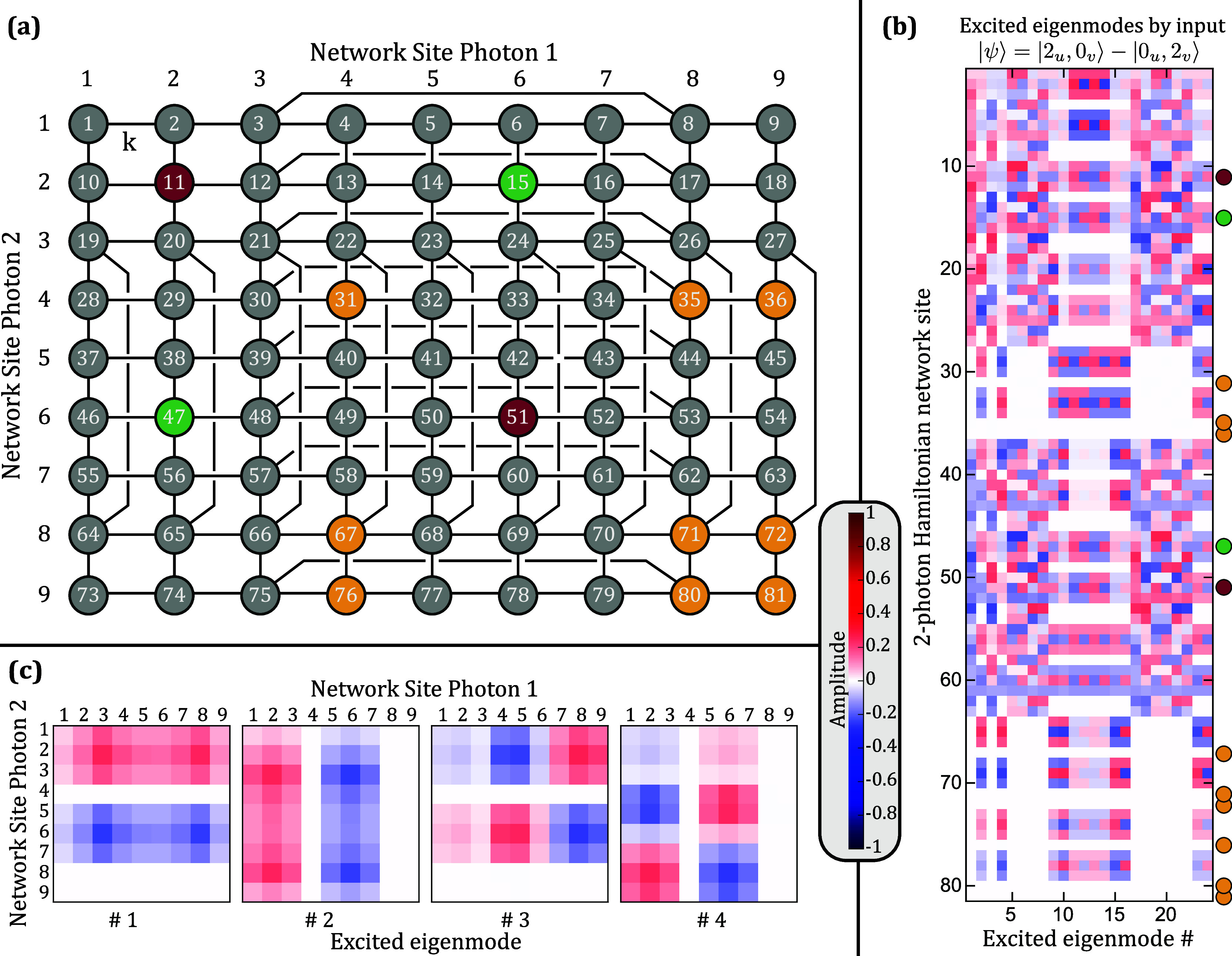
(a) Two-photon Hamiltonian **H**_Dist_ of the studied system can be represented by a 9 × 9 = 81 site graph. Each of its sites corresponds to the position of the two photons in the original graph at a time. There are now two latent symmetric pairs and nine singlet sites. (b) An antisymmetric two-photon excitation on site 11 and 51, |ψ⟩ = (|2_*u*_0_*v*_⟩ – |0_*u*_2_*v*_⟩)/ $\sqrt{2}$ , excites the 24 odd parity eigenmodes out of the total 81 eigenmodes. The amplitude on the singlet sites is zero for all of them, while both latent-symmetric pairs have odd parity amplitudes. An antisymmetric excitation on site 15 and 47 would excite the same modes. (c) The first four of the 24 eigenmodes excited by an antisymmetric excitation reshuffled to the shape of the 9 × 9 site graph.

Applying the same definitions as in the single-photon case, we study the evolution of an antisymmetric excitation, $|\psi_{1}\rangle =\left(|2_{u}0_{v}\rangle -|0_{u}2_{v}\rangle \right)/\sqrt{2}$ or $|\psi_{2}\rangle =\left(|1_{u}1_{v}\rangle -|1_{u}1_{v}\rangle \right)/\sqrt{2}$ , which correspond to exciting either site 11 and 51 or 15 and 47 of **H**_Dist_. Both cases exclusively populate the odd parity eigenmodes of the two-photon Hamiltonian **H**_Dist_ as shown in Figure 4b for |ψ_1_⟩.

These modes always have zero amplitude at the two-photon singlet sites. When exciting the latent-symmetric sites of **H**_Dist_ in an antisymmetric fashion, no correlations will be observed between the singlet sites of **H**: both photons will never be found at some combination of sites *a*, *b*, and *c*. However, correlations between singlet sites and nonsinglet sites of **H** do indeed occur: one photon can populate a singlet site *a*, *b*, or *c*, while the other photon is at a nonsinglet site. In Figure 4c, the first four of the excited modes are shown, reshuffled to the shape of Hamiltonian **H**_Dist_. The first mode is separable into photon 1 being in mode |λ_1_⟩ and photon 2 in mode |λ_2_⟩, as one would expect for distinguishable noninteracting particles. Furthermore, it has the same shape as the second one and the third as the fourth, just for the particles swapped.

### Indistinguishable Two-Photon Excitation

As a next step, we consider indistinguishable photons and study how latent symmetry affects the quantum interference of photons. In that case, the space of possible correlations decreases, since the outcome of the first photon being at site *m* and the second photon being at site *n* cannot be distinguished from the first photon being at site *n* and the second photon being at site *m*. Intuitively, the indistinguishable two-photon Hamiltonian **H**_Indist_ is obtained,^22^ by diagonally “folding” one-half of the Hamiltonian onto the other (Figure 5a). Specifically, the pairs of rows and columns that will become indistinguishable are added, the rows and columns that connect to two photons in the same mode, for normalization, are multiplied by $\sqrt{2}$ , and the constructed matrix is then divided by two. We thus end up with the ((*N* × *N*) + *N*)/2-site Hamiltonian (Figure 5b). An indistinguishable antisymmetric excitation $|\psi \rangle =\left(|2_{u}0_{v}\rangle -|0_{u}2_{v}\rangle \right)/\sqrt{2}$ again excites only the 12 odd parity eigenmodes of **H**_Indist_, which are zero at the singlet sites. Upon examination of the first two excited eigenmodes, it can be seen that the first one can be formed by individually folding either of the first two eigenmodes of distinguishable photon Hamiltonian, **H**_Dist_, over the diagonal onto itself. Similarly, the second mode can be formed by folding eigenmodes three and four in Figure 4c. As a result, we obtain half of the odd modes in total. While the latent symmetric site on the diagonal (both photons being found either at site *u* or *v*) remains unchanged, the parity in the eigenmodes of the second latent-symmetric pair (one photon being at site *u* and the other at *v*) causes destructive interference when folding the modes. As a consequence, the remaining site becomes a singlet site (green), with zero amplitude in the odd eigenmodes. The site, corresponding to correlations on sites *u* and *v*, thus also stays zero for all times as a result of the quantum interference of indistinguishable photons.

**Figure 5:**
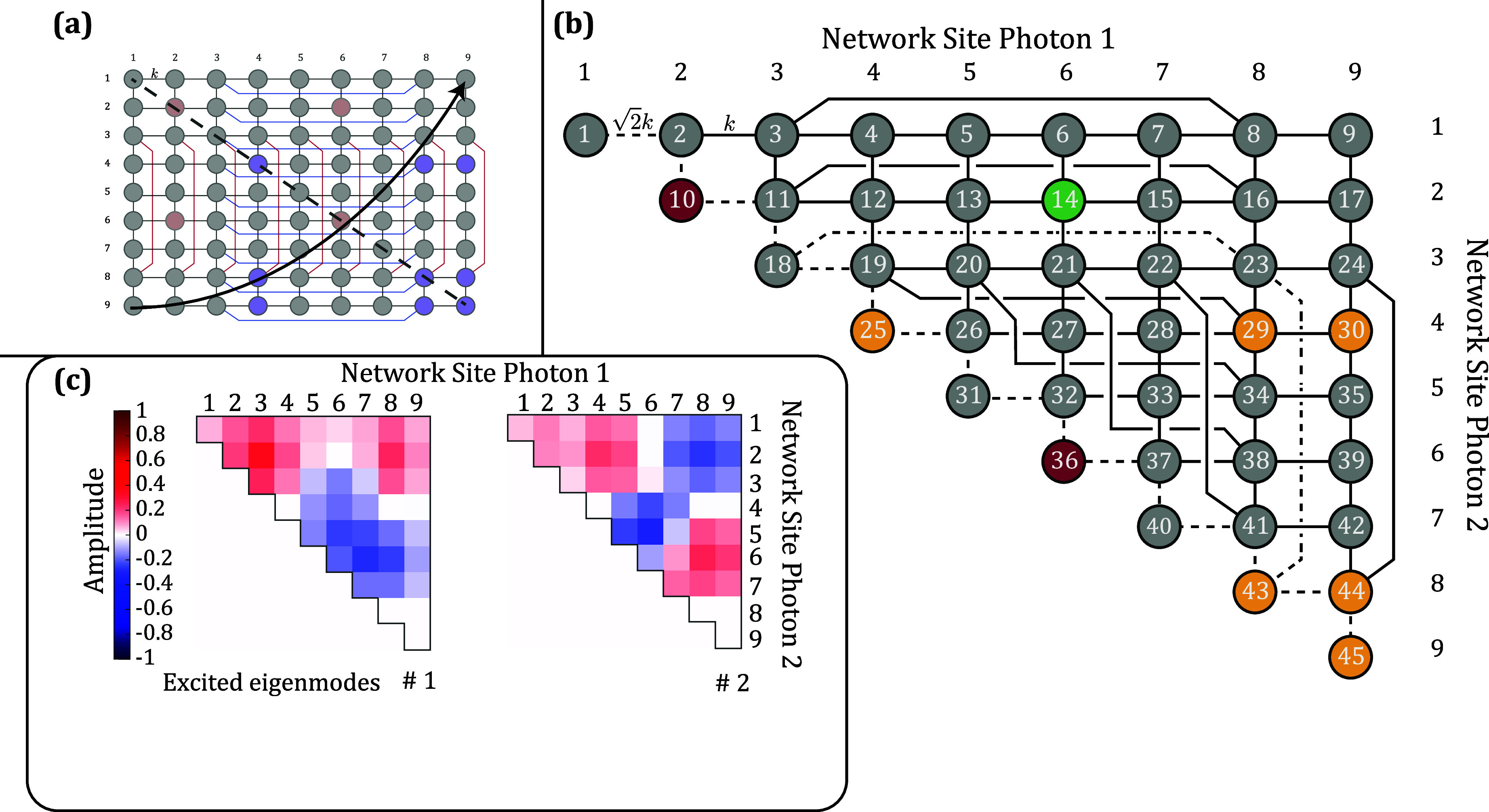
(a) Folding the two-photon Hamiltonian for distinguishable photons **H**_Dist_ across the diagonal yields the two-photon Hamiltonian or indistinguishable photons **H**_Indist_. (b) The two latent symmetric sites (red) as well as the single sites (yellow) on the diagonal remain constant. The off-diagonal singlet sites merge from six to three without changing their symmetry properties, while the off-diagonal latent symmetric sites (green) interfere destructively in the odd parity eigenmodes and become a singlet site. The dashed line indicates a coupling of $\sqrt{2}k$ , the solid line a coupling of *k*. (c) The first two out of 12 eigenmodes excited by an antisymmetric excitation $|\psi \rangle =\left(|2_{u}0_{v}\rangle -|0_{u}2_{v}\rangle \right)/\sqrt{2}$ reshuffled to the shape of the Hamiltonian, which can be directly constructed by “folding” the four eigenmodes shown in Figure 3c. As expected, singlet sites are zero, latent symmetric sites have odd parity.

## Conclusion

In conclusion, we theoretically studied the dynamics of coupled optical modes in a latent-symmetric system for single photons as well as for (in)­distinguishable two-photon excitations. It was demonstrated experimentally that antisymmetric single-photon excitations at the latent-symmetric sites are systematically precluded from populating singlet sites. Since the system can be arbitrarily extended at these sites without breaking the symmetry, this could be, e.g., used for the storage of information; an antisymmetric excitation would never leave the initial latent-symmetric system. We furthermore demonstrated theoretically that also all singlet sites of the constructed distinguishable two-photon Hamiltonian stay zero at all times. By extending this theory to the evolution of an indistinguishable two-photon excitation, we found quantum interference as a result of the transformation of two latent symmetric network sites to one singlet site. This way we contributed to a deeper understanding of latent symmetries and their influence on quantum light, which can be applied to implement, e.g., state transfer networks.

While we here used a network of evanescently coupled waveguides to illustrate the concept of latent symmetry, the concept itself is rooted in graph theory and can therefore readily be applied to any coupled-mode system in photonics such as nanophotonic metasurfaces and metamaterials, coupled resonators, or optomechanical systems. The eigenspectral domain offers an additional route for harnessing symmetries to control the behavior of photonic systems, without relying on real space or *k*-space, which furthermore connects well to the new approaches in non-Hermitian^14^ and topological physics.^15,23^ Moreover, latent symmetries provide a means to realize exciting new functionalities that go beyond what can be achieved with geometrically symmetric systems, for instance, by an extension to systems of which the eigenmodes are not simply related by parity on two latent-symmetric sites *u* and *v*, as is the case in this work, but with an additional scaling factor. Thus, latent symmetries add a powerful tool to design photonic metasurfaces with desired functionality for applications in photonic information processing, sensing, and metrology.^24,25^ It will be exciting to explore the interplay of latent symmetries and multiphoton quantum states, as it directly increases the dimensionality of the underlying graph. An excellent question would be whether it is possible to induce or suppress latent symmetries by introducing more photons and their (in)­distinguishability. That way, latent symmetries may support engineering quantum-photonic metasurfaces^26^ that shape the quantum behavior of light.

## Methods

The individual waveguides of the implemented network are inscribed by focusing 270 fs laser pulses from an ultrafast fiber laser amplifier (Coherent Monaco, wavelength 512 nm) at a repetition rate of 333 kHz through a 50× microscope objective (NA = 0.6) into fused silica (Corning 7980). The waveguide trajectories are defined by the motion of a high-precision translation stage (Aerotech ALS180). The photonic system was designed with an angle between adjacent waveguides of 30° to minimize undesired next nearest-neighbor couplings. Moreover, coupling characterization scans were performed to ensure that the diagonal and vertical couplings are identical. The respective distances were chosen as *x*_diag_ = 23.35 μm and *x*_vert_ = 23.1 μm, to establish a coupling value of *k* = 0.054 mm^–1^ for light with a wavelength of 814 nm. Waveguides *u* and *v* were connected to the front facet using a fanning section, to match the distance of the fibers in the incoupling fiber array (82 μm).

## Data Availability

The data sets generated and analyzed during the current study are available from the corresponding author on reasonable request.
